# Meaning-making coping under the threat of war: a qualitative study of people in Sweden

**DOI:** 10.3389/fsoc.2026.1917771

**Published:** 2026-10-13

**Authors:** Zahra Darvishpour, Fereshteh Ahmadi, Önver A. Cetrez

**Affiliations:** 1 Department of Sociology, University of Gävle, Gävle, Sweden; 2 Department of Psychology of Religion, Uppsala University, Uppsala, Sweden

**Keywords:** anticipated threat, faith-based coping, Meaning-Making Coping, qualitative research, RCOPE, secular existential coping, Sweden, war

## Abstract

**Introduction:**

The question of how individuals make meaning of, and cope with, the threat of war remains underexplored, particularly in highly secular societies and in contexts of anticipated rather than directly experienced conflict.

**Methods:**

This article reports findings from a qualitative study of meaning-making coping among people in Sweden, a country that has not experienced war within its borders for more than two hundred years but that now faces heightened geopolitical tension. The study forms part of the international research program Meaning-Making Coping with Crisis. Using a purposive sample of 41 adults in Stockholm and Gotland, data were collected through an online open-ended questionnaire and analysed thematically, informed by Pargament’s RCOPE framework and a socio-cultural model distinguishing faith-based from secular existential coping.

**Results:**

The findings show that both religious or spiritual and secular existential meaning-making strategies are present, but that secular strategies are markedly more prominent, while religious coping is peripheral and, where present, often confined to private practices such as prayer or to the church as a communal rather than devotional space. The secular existential strategies identified include relational, nature-based, cosmic, meditative, and artistic forms of meaning-making. A potentially novel form of secular existential coping, meaning-making through the absence of meaning, is also identified, in which psychological relief is achieved through the acceptance of human limitation and the relinquishing of ultimate meaning rather than its reconstruction.

**Discussion:**

Taken together, the results indicate that responses to the threat of war are multidimensional, reflecting an interplay of several meaning systems alongside emotional and cognitive processes, and that they are shaped by cultural, relational, and existential context rather than by religious belief alone. The study underscores the cultural, relational, and existential dimensions of coping with war-related threat and the need to extend established meaning-making and religious coping models to highly secular settings.

## 1 Introduction

The question of life’s meaning has long occupied a central place in human existence, particularly during periods of profound crisis such as war and armed conflict. Although the psychological consequences of war have been extensively examined, considerably less attention has been devoted to understanding how individuals cope with the threat and experience of war through meaning-making processes, both during and in the aftermath of such events.

The role of diverse cultural and ideological frameworks in shaping coping strategies requires further investigation, particularly with regard to differences between religious and non-religious individuals and variations across cultural contexts. This issue is especially important given the growing body of research indicating that religious affiliation and spiritual worldviews play a significant role in how individuals respond to and cope with crises (Fadilpašić et al., 2017; Gokani et al., 2023; Oviedo et al., 2022; Park et al., 2017; Schlechter et al., 2021).

Since 2013, our international research program, Meaning-Making Coping with Crisis, has brought together professors, associate professors, and researchers from Sweden and nine other countries to investigate coping strategies across a range of crisis contexts, including coping with cancer, bereavement following the loss of a child, the COVID-19 pandemic, and, more recently, war and armed conflict. This collaboration has resulted in four books and forty-seven peer-reviewed articles published in international journals. As part of this program, we have sought to examine meaning-making coping in relation to war across different cultural and political contexts. To this end, we have conducted qualitative studies in three countries affected by war in distinct ways: Ukraine, Sweden, and Iran. These studies explored how war and related geopolitical tensions influenced individuals’ coping strategies and meaning-making processes within their respective national contexts.

The study on which this article is based was designed to investigate meaning-making coping strategies among populations affected by war and conflict-related stress, focusing on both direct exposure to war and the anticipation of potential threats. Understanding how individuals navigate the psychological, existential, and social challenges associated with war is essential for the development of culturally sensitive interventions, mental health policies, and resilience-building initiatives. In the present article, we report only the findings from the Swedish study.

## 2 Theoretical framework

### 2.1 General coping with anticipatory threat

Sweden has experienced centuries of relative peace, and its population faces a passive anticipation of war. While not directly exposed to violence, heightened geopolitical tensions in Europe, particularly concerning Russia, have generated widespread public concern. Media coverage, government preparedness campaigns, civil defense initiatives, and cultural narratives of conflict contribute to heightened vigilance and anxiety.

Swedish coping strategies are generally characterized by a focus on uncertainty management and preparedness. Individuals rely on informational coping, risk assessment, and practical planning, while community and institutional frameworks provide reassurance and a sense of collective responsibility. Existential and reflective strategies, including ethical deliberation and prioritizing meaningful relationships, help maintain coherence and psychological equilibrium. However, relatively little is known about how people in Sweden make meaning of threats of war and cope with such threats on an existential level, especially considering that the country has not experienced war within its borders for more than 200 years. In this article, we focus on the meaning-making coping strategies revealed in our qualitative study in Sweden.

### 2.2 Important concepts

#### 2.2.1 Meaning-making coping

The study on meaning-making coping with war is of great importance, particularly within the broader international context of coping research in sociology and psychology. Meaning-making is described as “an important self-regulatory process within the individual, and a process of mutual regulation in relationships” (Davis and Hayes, 2011, p. 431). An individual’s sense of meaning in life lacks a clear and universally agreed-upon definition, while meaning-making processes are often complex and not easily identifiable (Park, 2010; Schnell, 2009). The current definitions are based on ambiguous and simple models that fail to take into account the complexity of the construct “meaning of life” (Martela and Steger, 2016). Despite the problem of exactly defining meaning-making, according to a psychological approach, there is some consensus on the importance of coherence, purpose, and significance (Lloyd, 2018).

#### 2.2.2 Faith-based coping

In our study, *faith-based coping* is understood as coping strategies that draw on beliefs, practices, or symbolic resources oriented toward a transcendent, sacred, or morally ordered reality. This includes both institutional religious practices (e.g., prayer, ritual participation) and non-institutional or personalized expressions of faith, interpreted as resources for existential meaning-making, ethical orientation, and emotional regulation rather than solely as manifestations of formal religiosity.

Despite using a single overarching concept, faith-based coping, to encompass both religion-oriented and spirituality-related coping in the present qualitative study, we found it necessary to address these approaches analytically. Previous qualitative studies on meaning-making and coping with war, as well as our other qualitative research on coping with various forms of crisis, consistently demonstrate that different existential orientations, religious, spiritual, and non-religious or secular, play a significant role in coping processes (la Cour and Hvidt, 2010; Ahmadi and Ahmadi, 2018; Park et al., 2017; van Uden and Zondag, 2016; Ahmadi et al., 2026).

In all our research concerning meaning-making coping, we have defined religion as a search for significance that unfolds within a traditional sacred context. It is then related to an organized system of belief and practice relating to a sacred source that includes individual and institutional expressions, serves a variety of purposes, and may play potentially helpful and/or harmful roles in people’s lives (Jenkins and Pargament, 1995, p. 52). Spirituality is defined as a search for connectedness with a sacred source that is related or not related to God or any religious holy sources (Ahmadi, 2006, pp. 72–73). Spirituality involves efforts to consider metaphysical or transcendent aspects of everyday life as they relate to forces, transcendent and otherwise. Spirituality encompasses religion as well as many beliefs and practices from outside the normally defined religious sphere.

At the same time, our methodological experience indicates that, in qualitative research, it is often difficult to distinguish religion-oriented and spirituality-oriented coping strategies in a reliable and valid manner. These orientations frequently overlap in practice and are not always clearly differentiated by respondents, particularly in non-institutional or culturally fluid contexts. Therefore, rather than separating religious-oriented, spiritual-oriented, and secular-oriented coping methods, we adopt a more analytically robust and psychometrically feasible distinction between *faith-based coping* and *secular existential coping*.

This choice is justified by both empirical and methodological considerations. Conceptually, the distinction captures meaningful differences in how individuals orient themselves toward transcendence, ultimate meaning, or moral frameworks during crisis, while avoiding artificial boundaries between religion and spirituality. Methodologically, it enhances construct clarity and supports internal consistency and content validity.

#### 2.2.3 Secular existential meaning-making coping

In many coping studies, the concepts of “religious” and “spiritual” have frequently been used to describe coping strategies grounded in existential concerns and aimed at meaning-making. However, in contrast to this dominant trend, several studies conducted over the past decades within our research program on *Meaning-making Coping with Crisis* across different countries (Sweden, China, South Korea, Turkey, Japan, Malaysia, Brazil, the Philippines, Iran, Portugal and Ukraine), as well as other related research (e.g., La Cour and Hvidt, 2010), have identified alternative coping strategies that cannot be categorized as either religious or spiritual. These strategies are more appropriately understood as existential in nature, involving a search for meaning that is not inherently connected to religion, religious symbols, or a clearly defined sacred source. Such strategies can be conceptualized as secular existential coping, emphasizing meaning-making processes that are unrelated to religion, sacred symbols, or spiritual authorities. The term is used here to denote individuals’ efforts to mobilize internal resources within the self, through relationships with others, and in engagement with the surrounding world in order to cope with crises that may give rise to experiences of existential emptiness.

To analyze these different forms of meaning-making coping, we developed a theoretical model based on our empirical findings (Ahmadi and Ahmadi, 2018). In contrast to some earlier research (La Cour and Hvidt, 2010), our model demonstrates that there is no conceptual overlap between secular and religious coping domains (Ahmadi and Ahmadi, 2018, p. 136). In this framework, secular meaning-making coping has no connection to traditional sacred contexts. Nevertheless, it may intersect with individuals’ attempts to connect with sources such as nature or the cosmos, which are not associated with God or conventional religious frameworks.

A profound sense of belonging, significance, and meaning in everyday life does not emerge in isolation but rather develops within broader cultural and social contexts. Accordingly, we assume that cultural settings play a decisive role in shaping the meaning-making coping strategies adopted by individuals in times of crisis. This assumption is grounded in our extensive work within an international research project conducted across 11 countries, in which we examined the cultural dimensions of coping (see Figure 1).

**Figure 1:**
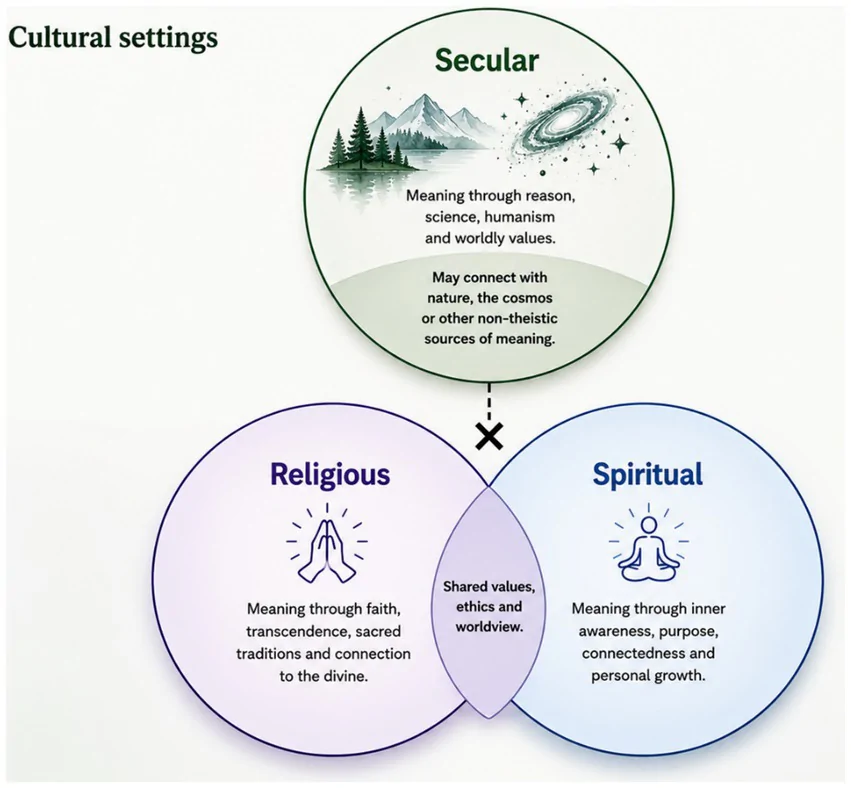
Alternative relation of existential meaning-making domains.

Through a series of qualitative and quantitative studies carried out in diverse national and international contexts and across different types of crises, we have both tested our initial hypothesis and developed a new theoretical model for the study of meaning-making coping (Ahmadi and Ahmadi, 2018). The present study forms part of this ongoing research program. Moreover, we aim to identify as wide a range as possible of coping strategies employed by individuals confronting various forms of crisis in different cultural environments. Such knowledge can provide health professionals, psychologists, and other practitioners with a more comprehensive understanding of coping resources that may be recommended to individuals in need.

### 2.3 Meaning-making coping and war

The significance of meaning-making coping in the context of war-related crises is illustrated in Figure 2. War introduces a range of profound challenges into individuals’ lives, including existential threats such as fear of injury, illness, or death (war-related crisis), as well as major disruptions to social relationships with family and friends and changes in occupational functioning among those directly affected by war-related attacks (social demands). These stressors have the potential to generate substantial negative psychological consequences. However, individuals’ coping efforts, shaped by personal resources such as resilience developed through previous crisis experiences, together with social, cultural, and other contextual resources, can strengthen their capacity to manage these challenges. The specific coping strategies adopted play a critical role in mitigating adverse psychological outcomes. Accordingly, personal, social, and cultural resources function as key mediators that influence how effectively individuals navigate crisis-related stressors (see Figure 2).

**Figure 2:**
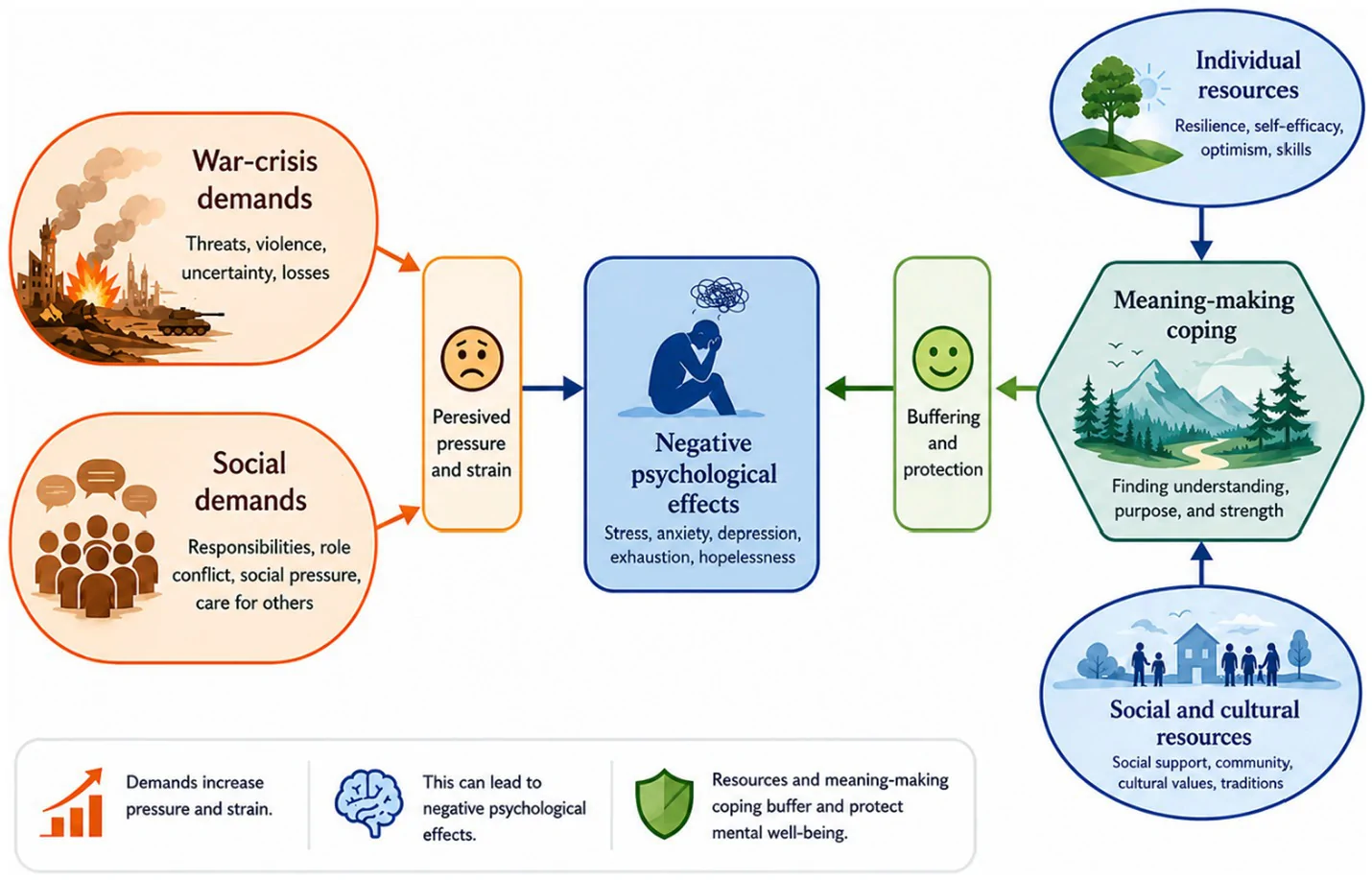
War-crisis demands and resources.

When confronted with stressful circumstances, individuals employ a range of coping strategies to reduce distress. Problem-focused coping aims to modify the conditions that cause or sustain the problem, whereas emotion-focused coping seeks to regulate emotional responses, often through behavioral or cognitive disengagement (Park et al., 2008). A third approach, meaning-focused coping, involves intrapsychic efforts to restore coherence by reconciling discrepancies between situational appraisals and individuals’ global meaning systems (Ahmadi et al., 2021; Ahmadi et al., 2022; Ahmadi and Zandi, 2021; Park, 2010; Zandi and Ahmadi, 2024). As noted by Park and Hanna (2022, p. 94), although problem-focused coping is often regarded as the most adaptive strategy, it is less applicable in low-control contexts such as trauma, loss, or severe illness. In such situations, meaning-making coping becomes particularly relevant and potentially more adaptive.

Research on highly stressful experiences, including serious illness, further underscores the importance of meaning and meaning-making coping (Moadel et al., 1999). Attempts to construct meaning in the face of adversity are associated with better psychological adjustment, particularly when individuals succeed in developing meaningful interpretations of their experiences (Park and Hanna, 2022).

### 2.4 State of the art

A substantial body of research has documented the psychological consequences of war, consistently linking exposure to armed conflict with elevated levels of Post-Traumatic Stress Disorder (PTSD), depression, anxiety, and prolonged grief among civilians and displaced populations (Steel et al., 2009; Charlson et al., 2019). Studies from diverse conflict settings, including Afghanistan, Bosnia, Syria, and Palestine, demonstrate how displacement, loss, and material deprivation undermine fundamental assumptions about safety, predictability, and control (Miller and Rasmussen, 2010; Tol et al., 2011). Research from Gaza further illustrates how continuous bombardment and infrastructural collapse intensify existential threat, fear, and psychological exhaustion (Aldabbour et al., 2026; Aqtam, 2025).

Within this literature, meaning-making has been conceptualized as a central coping process through which individuals integrate traumatic experiences into broader belief systems and restore coherence when core assumptions are disrupted (Park and Folkman, 1997; Park, 2010). Empirical studies among both civilians and veterans indicate that narrative reconstruction and existential interpretation of suffering facilitate psychological adaptation and endurance under prolonged threat (Larner and Blow, 2011; Currier et al., 2006). A strong sense of meaning in life has been shown to predict emotional stability in contexts of ongoing conflict (Russo-Netzer et al., 2025).

Worldview orientations, religious, spiritual, and secular, play a key role in shaping war-related meaning-making. Religious coping practices such as prayer and faith-based interpretation have repeatedly been identified as important resources for managing fear and uncertainty (Pargament, 1997; Ai et al., 2007), including in contemporary conflict zones such as Gaza (Aldabbour et al., 2026). At the same time, research has demonstrated that secular existential coping strategies, such as moral commitment, relational meaning, and community engagement, also contribute significantly to resilience and psychological stability in war-affected populations (Schnell, 2011; Ahmadi and Ahmadi, 2018). Coping is furthermore embedded in collective processes, including social solidarity, shared narratives, and communal support structures, which are central to sustaining meaning under prolonged threat (Hobfoll et al., 2007; Kirmayer et al., 2011).

Despite these insights, research on coping during the ongoing war in Ukraine has remained largely anchored in stress- and resilience-oriented psychological paradigms. Most Ukrainian studies conceptualize coping as functional adaptation aimed at emotional regulation and symptom reduction, with limited engagement with coping as an existential or cultural meaning-making process (Predko and Somova, 2022). Although religiosity, values, and volunteering are occasionally acknowledged, they are rarely analyzed as coherent systems of meaning through which individuals interpret suffering, loss, and uncertainty (Pyroh and Lytvynchuk, 2025). Secular existential meaning-making, particularly outside religious frameworks, remains especially under-theorized. Our studies on Ukrainians’ coping experiences have sought to address these gaps by exploring both religious and secular existential meaning-making strategies in the context of war (Melnyk et al., 2026; Ahmadi et al., 2026).

In Sweden, research addressing coping with war or anticipated war is sparse and primarily focused on refugees and military veterans. Existing studies document high levels of trauma-related symptoms and demonstrate the effectiveness of interventions such as Teaching Recovery Techniques (Sarkadi et al., 2018; Rondung et al., 2022; Durbeej et al., 2024), but rarely conceptualize coping in terms of existential meaning-making or societal interpretations of war.

Literature remains dominated by clinical and functional models of coping. There is a clear gap in theoretically grounded research that conceptualizes coping with war as a meaning-making process shaped by worldviews, particularly secular existential worldviews. This gap is especially evident in research on Sweden, where coping in relation to the anticipation of war has received little scholarly attention. Such anticipation constitutes a relatively new social phenomenon in a country that has not experienced war for more than 200 years. Addressing this gap is essential for advancing a more comprehensive understanding of how individuals and communities endure prolonged existential threats.

## 3 Materials and methods

### 3.1 Study design

This study employed a qualitative research design to explore meaning-making coping strategies in relation to anticipated war and crisis among individuals living in Sweden. Given the subjective and context-dependent nature of coping processes, a qualitative approach was considered appropriate for capturing participants’ perceptions and coping practices in depth.

### 3.2 Participants and sampling

A purposive sampling strategy was used to ensure variation in key characteristics, including age, gender, geographical location, worldview, and socio-political orientation. Eligible participants were adults (18 years and older) residing in Sweden who had experienced the effects of war either directly or indirectly.

Participants were recruited by disseminating study information and a survey link through housing associations, university websites, and selected cultural and political gatherings in Stockholm and Gotland.

Stockholm was selected due to its status as the capital and its socio-demographic diversity. Gotland was chosen because of its strategic importance and visible military presence, including Swedish forces and growing cooperation with international actors such as the United States. The presence of U.S. troops as part of NATO-related defence activities makes military preparedness more tangible in everyday life, allowing for comparison between a diverse urban setting and a region with heightened exposure to security and defence issues.

This approach enabled the inclusion of individuals from diverse professional and social backgrounds, including university faculty, students, administrative staff, and employees in both the public and private sectors.

A total of 41 participants completed the study. The sample was predominantly female (63.4%), with males representing 34.2% and one participant (2.4%) identifying as other. The age distribution was skewed toward older individuals, with 61.0% aged 50 years and above, 31.7% aged 35–50 years, and 7.3% aged 25–34 years. The relatively low participation of younger individuals may be related to generational differences in life experience and perceived relevance of war-related issues, which is further discussed in the Discussion section.

In terms of educational attainment, the majority of participants (80.5%) had completed university-level education, while smaller proportions reported upper secondary education (14.6%) or other post-secondary education (4.9%). This overrepresentation of highly educated individuals likely reflects common patterns in research participation, where individuals with higher education are more inclined to engage in academic studies. This should be considered when interpreting the findings, which primarily reflect the perspectives of a highly educated group.

Regarding marital status, 46.3% were married or in registered partnerships, while the remainder were single, divorced, cohabiting, or in other relationship arrangements.

Most participants reported an urban or semi-urban background, with 53.7% having grown up in large cities, 36.6% in small towns, and 9.8% in rural areas. (Supplementary Table 1: demographic data on participants).

### 3.3 Data collection

Data were collected through an online open-ended questionnaire designed to explore how individuals manage worry and anxiety in situations of crisis, particularly in relation to war or the threat of war.

The use of open-ended questions allowed participants to describe their experiences and coping strategies in their own words.

The survey was administered via the Sunet Survey platform at the University of Gävle. Data were collected between October and November 2025, during which all 41 participants completed the questionnaire.

### 3.4 Data analysis

The data were analyzed using thematic analysis following Braun and Clarke’s (2006) six-phase framework. The analysis combined an inductive exploration of participants’ responses with a theoretically informed interpretation drawing on meaning-making coping theory and Pargament’s RCOPE framework.

In the first phase, all responses were read repeatedly by the research team to become familiar with the material and to gain an overall understanding of how participants described their ways of coping with the threat of war. During this stage, preliminary notes and reflections were recorded regarding recurring patterns, meanings, and coping expressions.

In the second phase, the responses were coded line by line. Segments of text referring to coping resources, emotional regulation, existential reflection, religious practices, social relations, nature experiences, and other meaning-making processes were assigned initial codes. Coding remained data-driven at this stage to preserve participants’ own descriptions and minimise premature theoretical categorisation.

In the third phase, codes that appeared conceptually related were grouped into broader candidate themes. For example, codes referring to family support, contact with loved ones, and shared emotional reassurance were clustered under a preliminary relational coping category. Similarly, codes concerning experiences of forests, the sea, walking, and calmness in natural settings were grouped into a preliminary nature-based coping category.

In the fourth phase, all candidate themes were systematically reviewed against the original dataset. The research team assessed whether the coded extracts within each theme formed a coherent pattern and whether meaningful distinctions existed between themes. Themes that overlapped substantially were merged, while themes with insufficient supporting data were revised or discarded. This iterative process involved repeated movement between the coded material, the emerging thematic structure, and the theoretical framework.

Particular attention was given to the final determination of themes. In this stage, the researchers compared all candidate themes in relation to their conceptual boundaries, internal consistency, and theoretical relevance. Themes were retained only when they represented a distinct coping orientation supported by multiple data excerpts. Through this process, six overarching themes of secular existential meaning-making coping were finalized: relational coping, nature-based coping, cosmic meaning-making/unity of existence, meaning-making through absence of meaning, meditation, and art and music. Three themes associated with faith-based and religious coping were also identified: seeking spiritual support through prayer and scripture, religious community and institutional support, and church as a social and communal space.

In the fifth phase, each theme was clearly defined and named. Detailed thematic descriptions were developed to specify the central organizing concept of each theme and to distinguish it from related themes.

Finally, in the sixth phase, representative quotations were selected, and the themes were interpreted in relation to existing theories of meaning-making coping, secular existential coping, and religious coping.

To enhance analytic rigour, theme development was discussed repeatedly among the authors throughout the analytical process. Disagreements regarding coding or thematic categorization were resolved through discussion until consensus was reached. The final thematic structure was thus based on both empirical recurrence in the material and theoretical coherence with the study’s conceptual framework.

### 3.5 Ethical considerations

The study adhered to established ethical principles, including informed consent, confidentiality, and voluntary participation. Participants were informed about the purpose of the study and their right to withdraw at any stage.

Ethical approval for the overall project was obtained from the Swedish Ethical Review Authority (Reference No. 2015/126). All data were collected and stored securely, and participants’ integrity was ensured throughout the research process.

## 4 Results

Across the responses, several themes can be identified that shape how participants in this study cope with the threat of war. Taken together, these insights show that responses to the threat of war are not one-dimensional; rather, they reflect a complex interplay of multiple meaning systems alongside emotional and cognitive processes. A detailed overview of these themes is presented in Table 1.

**Table 1:** Overview of the coping themes identified in participants’ accounts of coping with the threat of war.

Religious/spiritual coping (RCOPE and non-religious spiritual coping)	Secular
Seeking spiritual support through prayer and scripture	Relational coping
Religious community and institutional support	Nature-based coping
Church as a social and communal space	Cosmic meaning-making/unity of existence
	Meaning-making through absence of meaning
	Meditation
	Art and music

From a theoretical perspective, these themes can be interpreted through Park’s meaning-making model of coping with stress (Park, 2010). This framework posits that when individuals encounter highly stressful or threatening situations, they attempt to reduce discrepancies between their broader systems of meaning and the interpretations they assign to specific crises. In doing so, they seek to restore coherence between global meaning, comprising fundamental beliefs, values, and existential assumptions, and situational meaning, or the appraisal of the immediate stressor (Park, 2010). Within this framework, participants in the present study appear to mobilize cognitive, emotional, and behavioral resources to re-establish psychological balance and coherence. The identified themes can therefore be understood as expressions of an ongoing process of meaning reconstruction under conditions of existential threat.

Additionally, drawing on Ahmadi’s (2006) and Ahmadi and Ahmadi’s (2018) framewor, these findings can be interpreted in terms of two broad orientations: faith-based coping, grounded in sacred or transcendent resources, and secular existential meaning-making. The results suggest that meaning-making is predominantly articulated through secular rather than explicitly religious frameworks. Although religion serves as a significant coping resource for some participants, many rely instead on a wider range of non-religious strategies when confronted with uncontrollable stressors associated with war.

The findings point to a limited role for religious coping, which appears peripheral rather than central in participants’ accounts. In contrast, secular forms of meaning-making are more prominent, often expressed through relational, natural, and existential perspectives. In many cases, religion is either absent or explicitly de-emphasized, while non-religious resources play a key role in shaping how individuals interpret and manage the threat of war. The following section elaborates on these themes, examining both religious coping and secular meaning-making in greater detail.

### 4.1 Religious coping

Using Pargament’s RCOPE framework (Pargament, 1997; Pargament et al., 2011), several religious coping methods can be identified in the material. These coping methods involve explicit references to God, religious beliefs, sacred practices, or religious institutions and authorities.

#### 4.1.1 Seeking spiritual support through prayer and scripture

##### 4.1.1.1 (RCOPE: seeking spiritual support)

One prominent theme concerns the use of prayer and religious texts as coping methods during periods of anxiety and distress (Pargament et al., 2011). Prayer is described as a means to seek comfort, inner peace, and emotional relief in the face of worry related to the threat of war. Reading the Bible is similarly framed as a source of reassurance and spiritual nourishment. These practices reflect a direct relationship with God and fit clearly within Pargament’s category of seeking spiritual support. This can, for example, be seen in the words of one participant, a 23-year-old man, when he says:

“Pray to God and in this way find peace of mind and soul. Read the Bible and its beautiful stories.”

Here, religion functions as an active coping resource that provides meaning, comfort, and emotional regulation.

#### 4.1.2 Religious community and institutional support

##### 4.1.2.1 (RCOPE: seeking support from clergy or congregation)

Another RCOPE consistent theme involves engagement with religious institutions and representatives (Pargament et al., 2011). Membership in a Christian congregation is described as a source of support and perceived benefit, while contact with priests or pastors is framed as a way of accessing knowledgeable guidance and assistance in difficult situations. This is illustrated, for instance, by one participant, an 88-year-old man, who states:

“I am a member of a Christian congregation and gain a great deal from it.”

In further support of this view, he also emphasizes the importance of relying on Christian clergy, saying:

“A Christian pastor or priest means that one can receive knowledgeable help in many situations.”

These accounts reflect reliance on institutional religious structures and authority figures, corresponding to Pargament’s emphasis on religious social and clerical support as coping resources.

#### 4.1.3 Church as a social and communal space: a non-religious spiritual coping

Some participants describe visiting churches despite not identifying as religious. In these accounts, the church is not primarily constructed as a sacred or doctrinal space but rather as an environment for calm, reflection, social connection, or aesthetic appreciation. This perspective is illustrated, for example, in the account of a 71-year-old woman:

“Spirituality, yes. I am not religious, but I enjoy visiting churches and beautiful cemeteries.”

A similar orientation is evident in another response:

“I am not particularly religious, but periods of unrest increase my inclination to visit a church once or twice a year.”

These narratives suggest a form of engagement that is cultural and experiential rather than explicitly religious, in which the meaning attributed to the setting is shaped by personal, emotional, and existential considerations rather than by theological belief. Consequently, such practices fall outside the scope of religious coping as conceptualized within RCOPE, despite taking place in religious environments. Instead, they may be more appropriately understood as expressions of spiritual coping, where the value of space lies in its capacity to facilitate reflection, emotional regulation, and a sense of continuity or connectedness.

### 4.2 Secular existential meaning-making coping

As explained before, Ahmadi and her research group identified coping methods among cancer patients, bereaved parents, and individuals affected by the COVID-19 pandemic and war that could not be categorized as religious or spiritual. These strategies were conceptualized within the framework of secular existential meaning-making coping, which includes subthemes such as cosmic meaning, experiencing life as part of a larger interconnected whole, and inner strength or personal power (Ahmadi, 2015; Ahmadi and Ahmadi, 2018). Meaning-making coping has been as mentioned before widely addressed in contemporary psychological research as a largely secular and cognitive–narrative process through which individuals attempt to re-establish coherence, purpose, and significance following stressful or traumatic experiences, typically by engaging in sense-making, benefit-finding, and the reconstruction of personal identity and life narratives.

In the present study, secular existential meaning-making coping is mainly observable along four pathways, all situated within a secular framework and unrelated to religious spirituality.

#### 4.2.1 Relational coping

One of the most significant themes across the dataset is the clear dominance of *relational coping*. In almost all responses, family, children, friends, and human relationships more broadly are identified as the most important sources of psychological security and emotional regulation in the face of the threat of war. In other words, relationality constitutes the core of the coping experience in this sample and shows considerably higher stability and recurrence compared to other coping methods. Within this framework, family and close relationships are not only perceived as sources of support, but also as a fundamental structure for maintaining psychological balance in conditions of crisis.

This finding is consistent with previous research showing that social support and close interpersonal relationships constitute central protective factors in situations of war, trauma, and forced uncertainty. Studies on war veterans and trauma survivors demonstrate that supportive family relations and meaningful social connections facilitate emotional reconciliation, resilience, and long-term coping with traumatic memories (Burnell et al., 2010). Research on military families has similarly shown that family cohesion and relational support function as key mechanisms of resilience under prolonged stress and exposure to conflict-related trauma (Saltzman et al., 2011). Comparable findings have also been reported in studies of refugee and war-affected populations, where family connectedness and interpersonal solidarity are described as essential resources for psychological survival and adaptation (Betancourt et al., 2012).

Participants in this study explicitly emphasize the importance of family and friends as central sources of support in coping with the war. When asked what had helped them to manage the situation, a 65-year-old female participant stated:

“Family and friends are crucial.”

A similar emphasis is evident in the response of an 88-year-old male participant, who noted:

“Yes, family is vitally important.”

The responses indicate that close interpersonal relationships play a multi-layered role in the coping process. First, they function as *a source of anxiety reduction and emotional comfort.* For instance, a 29-year-old female participant states:

“Access to loved ones is always important in order to engage in deeper conversations and to both give and receive comfort.”

This response highlights the importance of being in contact with close others in a way that allows for deep discussion and mutual exchange of comfort and reassurance. In other words, it points to both the emotional value of closeness (comfort and relief from anxiety) and its relational–cognitive value (opportunities for deeper reflection and shared understanding of the situation).

A second important function of relational coping is its role in reducing the perceived psychological distance from the crisis. Participants suggest that being with family makes external threats feel less immediate or more manageable. For instance, a 63-year-old male participant states:

“Being together makes everything else feel distant.”

In a similar vein, a 47-year-old male participant emphasizes:

“Yes; that we spend time together and do things that help us escape reality.”

These accounts indicate that relational coping not only provides emotional support but also reshapes how the crisis is cognitively and emotionally perceived, by creating a sense of distance from external stressors and facilitating temporary psychological relief.

A third function of this theme relates *to the enhancement of responsibility and perceived security through family relations.* In some responses, the presence of children and family members is directly associated with heightened concern, as well as with increased motivation for preparedness and protection. A 33-year-old participant (gender not specified) expresses this clearly:

“I’m just terrified for the sake of my children.”

Likewise, a 29-year-old female participant states:

“Losing my son in a war is one of my worst nightmares.”

These accounts illustrate how relationships with children can simultaneously function as a source of intense anxiety and as a driver of coping-oriented responses. In this sense, family ties generate both emotional vulnerability and a heightened sense of responsibility, shaping participants’ engagement with the ongoing threat.

Prior lived experiences appear to reinforce this pattern of relational orientation. For example, one participant explicitly draws on memories of past crisis experiences, linking them to the heightened importance of family connection:

“Yes, absolutely… it’s etched in my memory after the tsunami… a strong drive to stay in contact with family and to seek closeness.”

This account suggests that in situations of existential threat, such as war, participants turn to close interpersonal relationships as sources of stability, meaning, and emotional regulation. Relational coping emerges not merely as a form of social support but as a central framework for understanding and managing the crisis. Close relationships fulfill multiple functions, including fostering security, enabling meaning-making, and reducing perceived threat. Consequently, family ties and social networks can be understood as core resources shaping both the psychological and interpretive experience of crisis within this sample.

#### 4.2.2 Nature-based coping

Another prominent theme concerns the role of nature as a source of calm and emotional regulation. Participants consistently describe forests, the sea, natural silence, and walking as important elements in their coping processes, valued for their calming, grounding, and stress-reducing qualities. For several participants, being in natural environments not only helps them manage difficult periods but also opens onto broader processes of meaning-making. One participant, a 45-year-old male, states:

“I always turn to nature, the forest and the sea, when I go through difficult periods. In the forest, it is quiet, yet not entirely so; by the sea, I feel insignificant in comparison, which makes me appreciate what I have.”

Beyond its immediate regulatory role, the experience of the sea here introduces a moment of existential reflection, evoking awareness of human smallness in relation to the wider environment. This can be understood as a form of secular existential meaning-making, in which connection with nature fosters reflection on existence without necessarily involving spiritual interpretations. A 53-year-old participant makes this distinction explicit, differentiating between material and spiritual understandings of nature:

“In practical terms, yes. Nature is important for the survival of humanity and for our physical and mental well-being. Spiritually, no.”

For other participants, nature is bound up with cognitive distancing as much as with calm. A 39-year-old female participant explains:

“A walk, or sitting in nature, or near water or on a mountain makes me calmer. I sometimes go outside if nature is nearby and try to be in the present moment.”

Likewise, a 23-year-old male participant states:

“Nature can absolutely help, as I sometimes go for a walk outdoors and listen to music or a podcast to take my mind off things.”

Here nature supports coping both through presence and mindfulness in natural surroundings and, in combination with external stimuli such as music or podcasts, through the deliberate shifting of attention away from stressful thoughts. Taken together, these accounts indicate that nature functions primarily as a resource for emotional regulation, while for some participants it also becomes a site of existential meaning-making, prompting reflection on human existence, insignificance, and one’s place within a broader natural world. These two dimensions are not separate but interwoven throughout participants’ accounts.

#### 4.2.3 Cosmic meaning-making/Unity of existence

In this form of Existential meaning-making coping, participants perceive themselves as part of a “larger whole” and approach life from a philosophical perspective or a sense of the unity of existence. Nature, the universe, or existence functions as a framework for meaning construction. This view is explicitly expressed in responses. An 88-year-old male participant notes:

“Well, my life is part of something greater, yes!”

Similarly, a 58-year-old male participant emphasizes:

“Of course, life is part of something larger. I am just a small cog in a much bigger organic wheel.”

In some responses, this experience of being part of a larger whole is also linked to a sense of psychological security and inner stability. For example, a 63-year-old male participant explains:

“Yes, I feel that I am part of something greater, and that can provide perspective and a sense of security.”

Proceeding from this emphasis on human influence and responsibility, meaning is articulated primarily within a philosophical and ethical framework, where the individual is understood as an active agent shaping their life trajectory.

A 35-year-old male participant expresses this perspective as follows:

“From a humanistic perspective, human beings can exert influence, but life follows its course according to our choices.”

At the same time, this orientation can be interpreted as opening onto a broader, non-metaphysical sense of connectedness that resonates with notions of cosmic meaning or unity of existence. Here, the individual’s capacity to act is not seen in isolation, but as situated within a larger web of life, where human choices unfold in relation to natural, social, and existential conditions. Thus, even in the absence of explicit religious or metaphysical references, a form of existential unity emerges: the individual recognizes themselves as both influencing and being influenced by a wider reality. Meaning, in this context, arises through this dynamic interplay, where human agency is exercised within an interconnected cosmos, and where responsibility becomes a way of engaging consciously with one’s place in the broader continuity of existence.

#### 4.2.4 Meaning-making through absence of meaning

One interesting form of secular existential meaning-making coping we found in this study is meaning-making through the absence of meaning. Here, rather than experiencing connection to a larger whole, individuals emphasize human smallness, limitation, or even insignificance in the universe. This perspective is sometimes accompanied by a sense of determinism or lack of control over circumstances, for example:

“*I feel that everything is predetermined and I cannot influence it*.”

In other responses, a more explicit sense of existential insignificance appears:

“*…many times, I instead think about my insignificance*.”

This articulation can be interpreted as a form of secular existential coping grounded in the conscious decentring of the self. Rather than striving to construct affirmative or teleological meaning, the individual engages in a reflective reorientation in which the self is situated within a vastly larger and indifferent reality. Philosophically, this resonates with traditions of existential thought in which the recognition of human finitude and contingency does not necessarily produce despair but may instead open a space for a more lucid and unburdened mode of being. Meaning, in this framework, is not discovered as an objective structure nor constructed as a coherent narrative; rather, it is partially relinquished, and with it, the demand that existence must be justified. From a psychological perspective, this can be understood as a form of “negative” or minimal meaning-making, where adaptation occurs not through the amplification of purpose but through its attenuation. By acknowledging one’s relative insignificance and limited control, the individual reduces the cognitive and emotional strain associated with responsibility, uncertainty, and the need for coherence. This process may be seen as a variant of cognitive reframing, in which perceived threats are recontextualized within a broader existential horizon, thereby diminishing their subjective intensity. In this sense, the “absence of meaning” should not be understood as an absence of meaning-making itself. Rather, the recognition that events may lack an ultimate, predetermined, or human-centred meaning becomes part of the meaning-making process. Recontextualizing the self and the perceived threat within a broader existential horizon can itself generate a different form of coherence: not through discovering why events occur, but through accepting that such an explanation may be unavailable or unnecessary. Thus, what is relinquished is not meaning altogether, but the demand for an affirmative, purposive, or teleological meaning. It also bears resemblance to acceptance-based coping, where distress is regulated not by altering external circumstances or constructing compensatory beliefs, but by modifying one’s relationship to uncontrollable conditions. Importantly, this orientation does not imply nihilism in a pathological sense, nor what in the RCOPE framework is referred to as passive religious deferral, that is, a passive expectation that God will take control of the situation. Rather, it reflects a subtle shift from meaning as assertion to meaning as release. The individual does not deny the absence of ultimate meaning; instead, this absence itself becomes functional, allowing for a reduction in existential pressure and facilitating emotional regulation in the face of crisis. In this sense, “meaning-making through the absence of meaning” operates as a psychologically adaptive response, enabling a form of stability grounded in acceptance rather than resolution. Importantly, the absence of meaning did not refer to existential emptiness or meaninglessness. Rather, it referred to the absence of a specific explanatory, purposive, or personally relevant meaning attributed to suffering. Participants described finding peace not by discovering why suffering occurred, but by accepting that some aspects of existence may remain beyond explanation.

#### 4.2.5 Meditation

Meditation is widely described as a practice aimed at calming the mind, cultivating focused awareness, and promoting emotional regulation. Research shows that mindfulness and meditation techniques contribute to reduced anxiety, stress, and improved psychological well-being. At the same time, meditation involves observing thoughts and internal experiences, which facilitates reflective processes and increased self-awareness, including engagement with existential concerns and one’s sense of self. Although meditation has historical roots in religious traditions, it is now frequently practiced in secular contexts, where it is not necessarily associated with belief in God or adherence to a specific religious framework (American Psychological Association, 2019; Wang, 2025; National Center for Complementary and Integrative Health, 2022).

One of the informants (a 39-year-old woman) describes using meditation as a coping method as follows:

“I usually meditate when I am alone, which helps calm my thoughts about society and eases my anxiety quickly.”

Similarly, one 41-year-old male respondent notes:

“Meditation and spiritual intellectualizing … are part of my life and can intensify when I feel worried.”

From an RCOPE perspective, these practices cannot be straightforwardly categorized as religious coping. Instead, they represent religious or secular existential coping and would need to be treated as a separate sub-theme rather than subsumed under religious coping.

#### 4.2.6 Art and music

Finally, some participants describe music and art as important resources for coping, associated with grounding, emotional regulation, and bodily awareness. Although some of these experiences are framed in “spiritual” terms, participants explicitly distinguish them from religion, emphasizing their non-religious character. This is evident in the account of a 23-year-old male participant:

“I meditate often … I also listen to a lot of music to help me feel more grounded in my body.”

Similarly, a 63-year-old male respondent states:

“Religion, no. But there is certainly a sense of spirituality in art and music.”

These accounts suggest that music and art can function as forms of non-religious spirituality, where experiences of grounding, presence, and emotional regulation are expressed outside of institutional religious frameworks. This interpretation aligns with broader research showing that music can function as a versatile coping resource in situations of distress, crisis, and existential threat, operating across secular, spiritual, and religious frameworks. As previous studies suggest, musical engagement supports emotional regulation, affect modulation, bodily grounding, and the processing of difficult life experiences (Ruud, 1997; DeNora, 2000; Ahmadi, 2013; Ahmadi et al., 2016). Importantly, music is not inherently tied to a specific worldview; rather, its coping function depends on how it is experienced and interpreted by the individual.

In some contexts, as reflected in participants’ accounts, music operates as a secular coping strategy, offering psychological stabilization, bodily grounding, and a sense of continuity without reference to transcendence or the sacred. In other contexts, music may take on a more spiritual meaning, functioning as a medium for existential reflection, heightened awareness, or a sense of connection to something beyond the self. This dual positioning highlights how music can move fluidly across secular and spiritual domains, challenging rigid distinctions between religious and non-religious coping.

The findings illustrate how music and art exemplify the flexibility of coping processes. They demonstrate how the same practice can be experienced in different ways, ranging from embodied emotional regulation to spiritually framed meaning-making, depending on individual interpretation, cultural context, and worldview. In this sense, music operates as a key site where secular and spiritual forms of coping intersect rather than remain separate categories.

## 5 Discussion

The study shows that both religious/spiritual and secular existential meaning-making coping strategies are used by the informants in Sweden. However, secular coping strategies were more prominently present in the narratives.

Applying Pargament’s RCOPE framework to the gathered data helps identify patterns of religious coping, while also clarifying how participants draw on a wider range of meaning-making resources. The framework is particularly useful for capturing explicitly religious methods, such as prayer, engagement with scripture, reliance on clergy, and participation in religious communities. In these cases, coping is oriented around faith in God and reflects what Pargament describes as positive religious coping, where religion serves as a source of comfort, guidance, and emotional support.

At the same time, the material shows that many coping practices extend beyond these clearly religious forms. Participants frequently describe methods such as meditation, engagement with music and art, relationships with family and close others, and experiences in nature. While some of these are articulated in terms of spirituality or existential reflection, they are not grounded in explicit religious belief or reference to the sacred. Rather than being interpreted as religious coping, these practices point to broader processes of meaning making that coexist alongside, or independently of, religious frameworks.

Certain practices illustrate that coping cannot be understood solely in terms of observable behaviors but must be interpreted in relation to the meanings individuals attach to them. For example, music and meditation are experienced as spiritually meaningful by some participants while remaining detached from formal religion. Likewise, church spaces may serve as places of religious engagement for some individuals and as sources of tranquility, aesthetic appreciation, or social belonging for others. The significance of these practices, therefore, depends less on their form than on how they are experienced, interpreted, and incorporated into the coping process.

Several qualitative and quantitative studies on meaning-making coping in Sweden (Ahmadi and Ahmadi, 2018; Ahmadi and Zandi, 2021; Ahmadi et al., 2021; Ahmadi, 2015) indicate a similarly weak tendency toward religious coping among the population. From a cultural perspective, this limited reliance on religious coping can be interpreted as reflecting broader patterns of secularization and the marginal role of religion in everyday life.

First, Sweden is commonly described as one of the most secular societies in the world. While a majority of the population formally belongs to the Church of Sweden, levels of belief in God, participation in religious practices, and the perceived importance of religion in everyday life are generally low. Religion is often seen as a private or cultural matter rather than a central framework for interpreting life events. As a result, when individuals face stress or crisis, they are less likely to turn to explicitly religious resources such as prayer, clergy, or scripture, which form the core of religious coping in frameworks like RCOPE.

Second, secularization in Sweden has been accompanied by strong trust in institutional and welfare systems. The extensive welfare state provides social, economic, and healthcare support, reducing reliance on religious institutions as sources of comfort or assistance. In this context, coping is more often embedded in psychological, social, and institutional resources rather than religious ones. People may turn to professional support, social networks, or self-help strategies rather than faith-based practices.

Third, cultural norms in Sweden tend to emphasize individual autonomy, rationality, and emotional self-regulation. This aligns with coping strategies that are relational, reflective, or nature-based rather than explicitly religious. Practices such as spending time in nature, engaging in creative activities, or relying on close relationships are more culturally normalized ways of dealing with stress and existential concerns. Even when individuals describe experiences in terms of “spirituality,” this is often non-theistic, individualized, and detached from organized religion.

Moreover, the findings of the present study are consistent with previous research emphasizing the importance of meaning-making coping in situations characterized by low control, uncertainty, and existential threat (Park, 2010; Park and Hanna, 2022). As earlier studies have shown, when individuals are confronted with crises such as war, trauma, severe illness, or loss, coping processes often shift from problem-focused strategies toward meaning-focused and existential forms of coping, through which individuals attempt to restore psychological coherence and regulate emotional distress (Ahmadi et al., 2021; Ahmadi et al., 2022; Ahmadi and Zandi, 2021; Zandi and Ahmadi, 2024). In line with these theoretical perspectives, the participants in the present study relied predominantly on relational, existential, emotional, and nature-based resources rather than on attempts to directly control or change the threatening situation itself.

At the same time, the present findings extend previous meaning-making research by identifying a potentially new form of secular existential coping, conceptualized here as “meaning-making through the absence of meaning.” Unlike traditional models of meaning-making, which often assume that individuals cope by constructing coherence, purpose, or existential significance, this coping orientation involves the partial relinquishing of the need for ultimate meaning itself. Participants expressing this orientation emphasized human limitation, insignificance, or lack of control within a larger and indifferent reality and appeared to achieve psychological relief through acceptance rather than through the reconstruction of meaning. In this sense, adaptation emerged not through the amplification of meaning, but through a reduction of meaning-related demands and existential expectations.

This finding may contribute to further theoretical development within the field of meaning-making coping, particularly in secular contexts where existential adaptation may occur independently of religious belief, transcendence, or coherent existential narratives. It also suggests that coping in situations of uncontrollable crisis may involve not only the reconstruction of meaning, as emphasized in previous research, but also the acceptance of ambiguity, uncertainty, and the absence of ultimate meaning as psychologically adaptive responses.

The prominence of relational coping in the present study can also be situated within broader theoretical developments in meaning-making coping research. Previous work has consistently highlighted the importance of social and relational resources in coping with adversity, particularly in the context of trauma, loss, and existential crisis (Folkman and Moskowitz, 2000; Bonanno, 2004; Neimeyer, 2019). Within meaning-making frameworks, relationships are increasingly understood not only as sources of emotional support, but also as central contexts in which individuals construct coherence, purpose, and significance in response to stressful life events (Park, 2010; Park and Hanna, 2022). This perspective underscores the role of relational embeddedness in meaning-making processes, particularly under conditions of existential threat.

In addition, research on secular existential coping has demonstrated that meaning-making processes are often embedded in everyday relational contexts, where individuals draw on family, friends, and close interpersonal ties as key resources for maintaining psychological stability and existential orientation (La Cour and Hvidt, 2010; Ahmadi and Ahmadi, 2018). Within this perspective, relational coping is not limited to providing emotional support but also functions as a key site for the construction and maintenance of meaning under conditions of uncertainty and existential threat. Taken together, these perspectives suggest that relational coping should be understood as an integral dimension of meaning-making processes rather than solely as a form of social support.

Although the importance of social support in coping processes has been well documented across a range of studies and cultural contexts, including research conducted in China and other non-Western societies (Ahmadi et al., 2016; Ahmadi et al., 2017; Ahmadi and Ahmadi, 2018; Ahmadi et al., 2019; Pennebaker and Harber, 1993; Ke et al., 2010), the present findings indicate that this pattern is also strongly present in the Swedish context. This is particularly noteworthy from a cultural perspective, as Sweden is often characterized as a highly individualistic society with a strong orientation toward personal autonomy and independence. Despite this, the present data consistently show that participants identify family, children, and close relationships as their most important sources of psychological security, emotional comfort, and even meaning in the context of crisis.

This pattern can be understood within broader processes of social transformation in Sweden, a society that has traditionally been described as one of the most individualistic cultures in the world (Hofstede, 2001; Daun, 1996). However, contemporary research suggests that Swedish social and family structures have undergone significant transformation in recent decades due to increasing migration, cultural diversification, and processes of globalization (Castles et al., 2014). These developments have contributed to changes in patterns of social relations, including forms of interpersonal connectedness, family organization, and social dependency, which are increasingly being reconfigured in contemporary Swedish society. Within this context, family and close relationships continue to play a central role as sources of psychological security and meaning, even within a setting historically associated with strong norms of individual autonomy. This observation aligns with perspectives suggesting that processes of individualization in modern societies do not necessarily lead to the weakening of close relationships; rather, they may give rise to new and reconfigured forms of emotional and relational interdependence (Beck and Beck-Gernsheim, 2002; Berggren and Trägårdh, 2006).

Accordingly, the prominence of relational coping in the present study can be understood not only as a universal human pattern, but also as a contextually shaped and evolving form of meaning-making in contemporary Swedish society, which may in part be linked to generational changes and shifting attitudes toward family and friendship in Sweden. Empirical observations and sociological analyses increasingly suggest a shift toward more positive and relational attitudes toward family and friends, particularly among younger generations. These relationships are now more frequently understood as crucial sources of support, security, and resilience, especially in times of social and economic uncertainty.

A central explanation for this shift lies in the restructuring of the Swedish welfare state. From the early 1990s onward, Sweden has undergone economic crises, fiscal consolidation, and a gradual marketization of welfare services. Privatization and New Public Management reforms in sectors such as healthcare, education, and eldercare have altered the conditions under which citizens access public support. While the welfare state remains comparatively strong, its role as a universal and comprehensive safety net has been partly weakened. Therefore, individuals increasingly face situations in which public assistance is limited, delayed, or insufficient. In this context, family members and close friends have emerged as vital complements to formal welfare institutions, providing practical, emotional, and financial support during periods of unemployment, illness, or life transitions.

Generational differences are particularly evident when comparing cohorts socialized under different welfare regimes. Older generations, especially those who grew up during the post-war expansion of the welfare state, internalized norms of self-sufficiency and independence from family in adulthood. Living alone and minimizing reliance on relatives were seen as markers of autonomy and successful integration into modern society. In contrast, younger adults exhibit more favorable attitudes toward interdependence. Among these cohorts, seeking and offering support within family and friendship networks is increasingly normalized and morally legitimized. This development reflects not a rejection of individual autonomy, but rather a reconfiguration in which autonomy coexists with strong relational commitments.

Immigration and increasing cultural diversity constitute another key factor shaping changing attitudes toward family and social relations. Over recent decades, Sweden has become home to significant numbers of immigrants originating from collectivist or group-oriented societies, where family solidarity and intergenerational support play a central role. In these contexts, family is often understood as a primary source of identity, obligation, and social protection. Such cultural models contrast with, but also interact with, the historically dominant Swedish emphasis on individualism and state-based support. Importantly, these alternative normative frameworks have not remained confined within immigrant communities but have diffused into broader social contexts.

In addition to contextual explanations, generational differences may also help account for lower participation among younger respondents (25–34 years). Younger cohorts have not been directly exposed to war to the same extent as older generations, and, as Mannheim’s theory of generations suggests, such formative experiences shape how individuals relate to social issues. The absence of lived experience may therefore reduce the salience of war and limit engagement with related topics (Mannheim, 1952; Pilcher, 1994; Ramirez and Haas, 2021).

Psychological research further indicates that younger individuals often perceive and engage with risk differently, sometimes viewing distant or abstract threats as less personally relevant, which may reduce their motivation to participate (Kim et al., 2018). In addition, life-course factors play a role: younger individuals are less likely to have established families or dependents, which can lower the perceived immediacy of security-related concerns such as war. Together, these factors may contribute to the lower participation observed in this age group.

Finally, it is important to note that one possible explanation for the relatively low number of younger informants in this study may be related to broader shifts in the political and cultural landscape in Sweden. As indicated by exit polls from the 2022 general election, younger voters did not cluster around a single political orientation but showed relatively strong support for right-leaning parties, particularly the Moderate Party (M) and the Sweden Democrats (SD), with the latter receiving around 22 percent of the vote among the youngest age group. This development suggests that right-wing perspectives have gained increased resonance among younger cohorts. Given that right-wing and especially right-wing populist discourses in Sweden have often emphasized issues such as national security, crime, and external threats, they may also contribute to a normalization of war-related concerns within public debate. In such a context, war may be framed less as an exceptional existential crisis requiring active coping and more as a background condition of geopolitical reality.

This potential normalization may, in turn, influence how younger individuals relate to the topic of war and their willingness to reflect on or articulate coping strategies. If war is perceived as distant, abstract, or already integrated into everyday political narratives, the perceived need to engage in explicit coping processes may be reduced. Consequently, younger individuals may be less inclined to participate in studies addressing coping with war, or may find such questions less immediately relevant to their lived experience. While this interpretation remains speculative, it points to the importance of considering generational and political context when interpreting the composition of the sample, as well as how broader ideological trends may shape not only attitudes toward war, but also the salience of coping as a subject of reflection.

Future research could further examine how meaning-making and coping strategies vary across demographic groups, particularly between women and men and across different age groups. While the present study provides some indications of generational differences, its qualitative design and sample do not allow for systematic comparisons between these groups. Future studies with larger and more diverse samples could therefore investigate whether gender, age, and life-course position shape the use and interpretation of relational, nature-based, existential, and religious coping strategies. Such research could also help clarify whether the coping orientations identified in the present study are broadly shared or are shaped by particular demographic and social experiences.

In conclusion, this study highlights the complexity and contextual nature of coping processes in contemporary Swedish society, where secular, relational, and existential forms of meaning-making play a more prominent role than explicitly religious strategies. By situating the findings within both the RCOPE framework and broader meaning-making theories, the analysis demonstrates that coping cannot be reduced to predefined categories but must be understood in relation to cultural context, individual interpretation, and lived experience. The identification of emerging forms such as “meaning-making through the absence of meaning” further suggests the need to expand existing theoretical models to better account for adaptation in highly secular settings. The study contributes to ongoing discussions on coping by underscoring the importance of integrating cultural, relational, and existential dimensions into analyses of how individuals respond to crisis, uncertainty, and existential threat.

## Data Availability

The raw data supporting the conclusions of this article will not be available due to ethical concerns. However, coded and pseudonymised data can be made available upon request.
