# Supplementary material for: Meaning-making coping under the threat of war: a qualitative study of people in Sweden
Source: Front Sociol. 2026 Oct 13;11:1917771. doi: 10.3389/fsoc.2026.1917771 (PMC13710049; doi:10.3389/fsoc.2026.1917771)
Supplement: Supplementary file 1 [file Table_1.DOCX]

Supplementary Material

# 1 Supplementary Tables

Supplementary Table 1. Demographic data of participants.

| **Variable** | **Category** | **Number of Respondents** | **Percentage (%)** |
| --- | --- | --- | --- |
| **Gender** | Female | 26 | 63.4 |
|  | Male | 14 | 34.2 |
|  | Other | 1 | 2.4 |
| **Age Group** | 25–34 years | 3 | 7.3 |
|  | 35–50 years | 13 | 31.7 |
|  | 50+ years | 25 | 61.0 |
| **Highest level of education completed: Number of responses** | Primary school (or equivalent compulsory education) | 0 | 0 |
|  | Upper secondary school | 6 | 14.6 |
|  | Post-secondary education (not  college/university) | 2 | 4.9 |
|  | Univeristy or college | 33 | 80.5 |
| **Marital status** | Single/divorced/seprated | 9 | 22 |
|  | Living apart (in a relationship) | 4 | 9.8 |
|  | Cohabiting part (Sambo)ne | 6 | 14,6 |
|  | Married/register partnership | 19 | 46.3 |
|  | Other | 3 | 7.3 |
| **Childhood Environment of the Informants** | Big city | 22 | 53.7 |
|  | Small Town | 15 | 36.6 |
|  | Rural area | 4 | 9.8 |
